# Pharmacological Treatment of MASLD: Contemporary Treatment and Future Perspectives

**DOI:** 10.3390/ijms26136518

**Published:** 2025-07-07

**Authors:** Krzysztof Drygalski

**Affiliations:** Department of Hypertension and Diabetology, Medical University of Gdansk, ul. Mariana Smoluchowskiego 17, 80-214 Gdańsk, Poland; drygalskikrzysztof@gmail.com

**Keywords:** MASLD, NAFLD, MASH, NASH, treatment

## Abstract

Metabolic dysfunction-associated steatotic liver disease (MASLD), formerly NAFLD, is the most prevalent chronic liver disease worldwide. Strongly linked to obesity, type 2 diabetes, and metabolic syndrome, MASLD poses a growing health burden. Despite its high prevalence and risk of progression, no pharmacological treatment is currently approved. This narrative review provides an overview of emerging pharmacological treatments under clinical investigation, with a particular focus on agents recently evaluated in randomized clinical trials. A systematic search of the ClinicalTrials.gov database through to April 2025 was conducted to identify relevant studies. Investigational drugs were categorized by their molecular mechanisms, and data on efficacy, safety, and clinical development phases were summarized. The most extensively studied drug classes include GLP-1 receptor agonists, PPAR agonists, and FXR agonists, as well as inhibitors of ACC and DGAT. These therapies have shown promising effects on hepatic steatosis, liver enzyme levels, and metabolic markers and may be introduced into clinical practice in the near future.

## 1. Introduction

Metabolic dysfunction-associated steatotic liver disease (MASLD), formerly known as non-alcoholic fatty liver disease (NAFLD), is the most common chronic liver disease worldwide, with an estimated prevalence affecting approximately 25–30% of the global population [1]. The prevalence is significantly higher among individuals with obesity, type 2 diabetes mellitus (T2DM), and other cardiometabolic risk factors constituting metabolic syndrome [1]. The recently redefined terminology from NAFLD to MASLD underscores the central role of metabolic dysfunction in disease pathogenesis, offering a clearer and more inclusive framework for diagnosis and management [2].

MASLD is characterized by hepatic steatosis, defined as triglyceride accumulation in more than 5% of hepatocytes, and may progress to steatohepatitis and hepatic fibrosis if left untreated. In a subset of patients, this progression can lead to cirrhosis and, more rarely, hepatocellular carcinoma (HCC) as a late-stage complication [2]. Nevertheless, hepatocyte steatosis remains the central point of the disease, affecting liver function through multiple molecular pathways [3]. The condition is typically asymptomatic in its early stages and frequently discovered incidentally during imaging studies or blood tests performed for unrelated reasons. This often leads to delayed diagnosis, by which time the steatosis has been silently progressing for months or years, subtly impairing aspects of metabolic homeostasis such as glycemic control and lipid profiles [4].

**Figure 1:**
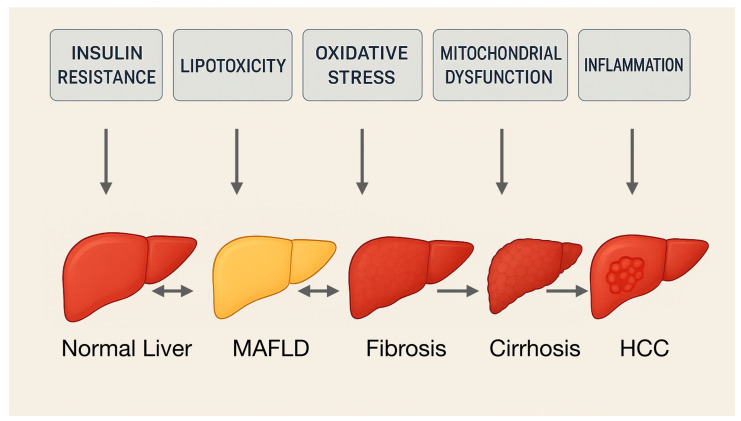
Progression of MAFLD according to the “multiple-hit” theory.

The pathophysiology of MASLD is complex and multifactorial. Insulin resistance is a central mechanism, promoting increased adipose tissue lipolysis and hepatic influx of free fatty acids. These metabolic alterations lead to hepatocellular lipid accumulation and subsequent lipotoxicity, oxidative stress, mitochondrial dysfunction, and inflammation [5,6,7]. On the other hand, lipid accumulation in hepatocytes itself promotes hepatic insulin resistance. Further disease progression from simple steatosis involves an interplay of various metabolic disturbances. This is best captured by the “multiple-hit” theory, which posits that liver steatosis and its progression to steatohepatitis, and fibrosis result from the cumulative, and often simultaneous, impact of several pathogenic factors (Figure 1) [8,9]. Beyond steatosis and lipotoxicity as the first “hit”, other “hits” include oxidative and endoplasmic reticulum stress, mitochondrial dysfunction, and alterations in the gut–liver axis—particularly dysbiosis and increased intestinal permeability that facilitate translocation of bacterial endotoxins [10,11]. These stimuli collectively activate hepatic immune responses, perpetuate chronic inflammation, and trigger fibrosis. Genetic predispositions like polymorphisms in PNPLA3 or TM6SF2 genes further modulate individual susceptibility and disease course [12,13]. This multifactorial model underscores the heterogeneity of MASLD and highlights the need for comprehensive management strategies that extend beyond simple lipid reduction to include modulation of inflammation and oxidative stress, body weight management, or glycemic control.

Aside from liver-specific consequences such as cirrhosis and HCC, MASLD carries significant systemic risks. It is now recognized as a major contributor to morbidity and mortality through its strong association with cardiovascular diseases—the leading cause of death worldwide—as well as obesity, T2DM, and chronic kidney disease [1,2]. In fact, MASLD is the leading cause of liver transplantation in women and the second leading cause, after alcohol-related liver disease, in men in the United States [14].

**Figure 2:**
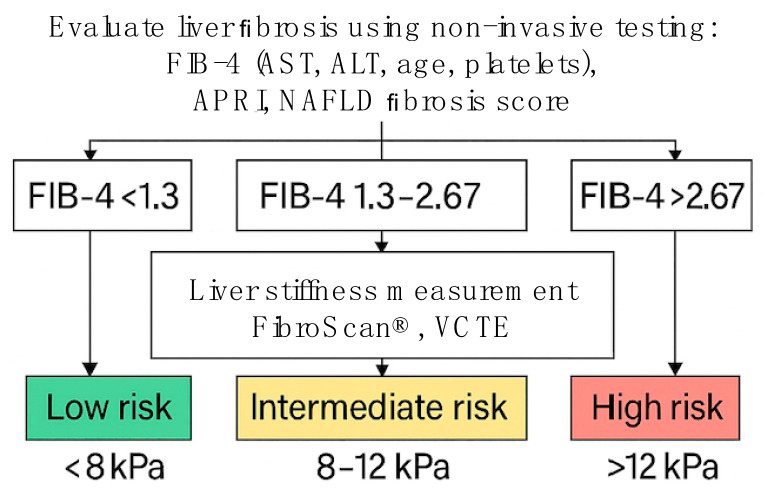
Non-invasive evaluation of liver fibrosis risk.

The diagnosis of MASLD is based on imaging evidence of hepatic steatosis in individuals with metabolic risk factors, following the exclusion of other potential causes of liver fat accumulation. Ultrasonography is the most widely used screening tool, owing to its broad availability and low cost; however, its sensitivity is limited, particularly in detecting mild steatosis. More advanced imaging techniques, such as transient elastography (e.g., FibroScan) and MRI-based modalities, provide greater sensitivity and the added benefit of assessing liver stiffness [2,15,16]. Although liver biopsy remains the gold standard for diagnosing steatohepatitis and staging fibrosis, its use is generally reserved for specific clinical situations due to its invasive nature. To assist in the non-invasive evaluation of liver fibrosis and guide treatment strategies, clinical scoring systems like FIB-4, along with transient elastography, have been developed to facilitate risk stratification (Figure 2) [2].

Given the high global burden, complex pathophysiology, and systemic impact of MASLD, effective therapeutic strategies and clear treatment recommendations are urgently needed. While lifestyle modification remains the cornerstone of management, long-term adherence is often challenging, and no pharmacological therapies have yet received widespread regulatory approval for MASLD. However, in March 2024 the FDA approved resmetirom, an oral thyroid hormone receptor-β (THR-β) agonist, in combination with diet and exercise for the treatment of adults with non-cirrhotic non-alcoholic steatohepatitis (NASH) with moderate to advanced liver fibrosis (consistent with stage F2 to F3 fibrosis) [17]. Nevertheless, there is still no approved treatment for MASLD/NAFLD itself.

In recent years, there has been growing interest in developing new drugs that target the key molecular mechanisms driving the disease. Numerous randomized clinical trials (RCT) have evaluated novel agents with various mechanisms of action, ranging from insulin sensitizers and lipid metabolism modulators to anti-inflammatory and antifibrotic compounds. In this review, I summarize the current treatment landscape of MASLD and critically assess recent clinical trial data to highlight emerging pharmacological options that may soon become part of routine clinical practice.

## 2. Contemporary Treatment Recommendations

Effective management of MASLD primarily targets the underlying metabolic disturbances driving disease progression. Lifestyle intervention—particularly achieving weight loss through tailored dietary adjustments and regular physical activity—remains the foundation of treatment [18]. Evidence shows that even modest reductions in body weight can lead to significant improvements in liver fat content, inflammation, and fibrosis. Importantly, the focus is not on rapid or drastic weight loss, but rather on a gradual, sustainable approach that promotes long-term health benefits. A steady reduction of approximately 0.5 to 1 kg per week is generally recommended, with an initial goal of losing 5–10% of baseline body weight over the course of three to six months [19,20]. This weight should ideally be maintained for a similar period before pursuing additional gradual reductions if necessary [13,16]. Despite the proven benefits, maintaining these lifestyle changes can be difficult in real-world settings, particularly among individuals with comorbidities [15]. Consequently, there has been growing interest in adjunct pharmacological treatments. The following sections will review the available evidence on physical activity, dietary approaches, and emerging pharmacotherapies in the treatment of MASLD.

## 3. Dietary Interventions in MASLD

Dietary modification remains a fundamental component in the management of MASLD, with strong evidence supporting its role in reducing hepatic steatosis, improving metabolic parameters, and preventing disease progression. The effect of dietary interventions and lifestyle changes in MASLD has been profoundly reviewed recently [21]. Given that excess caloric intake, insulin resistance, and poor diet quality contribute significantly to intrahepatic fat accumulation, dietary changes aimed at creating a caloric deficit are central to treatment. Most guidelines recommend a hypocaloric diet with a daily energy deficit of 500–1000 kcal, targeting gradual, sustainable weight loss [22,23]. A reduction of 5–10% in body weight over 3–6 months has been shown to yield significant improvements in liver fat, insulin sensitivity, and inflammation, with weight maintenance or further reduction as the next step.

Among the dietary patterns studied, the Mediterranean diet is the most consistently recommended by international guidelines (EASL-EASD-EASO, ESPEN, and APASL), owing to its high content of monounsaturated and polyunsaturated fats, fiber, antioxidants, and polyphenols [13,22,23,24]. It has demonstrated efficacy in reducing liver fat, improving glycemic control, and lowering cardiovascular risk [25,26]. Other dietary strategies with potential benefits include high-protein diets—especially those emphasizing plant-based proteins—and low-carbohydrate ketogenic diets, which may improve insulin sensitivity and reduce liver fat content [27,28,29]. Intermittent fasting, which focuses on meal timing rather than content, has also shown effects in reducing hepatic steatosis and improving metabolic markers, though comparative data with other diets are limited [30,31,32,33].

Ultimately, dietary treatment must be individualized to patient preferences, metabolic status, and comorbid conditions. The key to success lies in achieving and maintaining a negative energy balance through a sustainable, healthy diet. While the Mediterranean diet currently offers the strongest overall evidence base, flexible adaptation of its principles to accommodate individual needs appears to be the most practical and effective approach in clinical practice.

## 4. Physical Activity and Lifestyle Modifications in MASLD

Lifestyle modification, particularly increased physical activity, plays a pivotal role in the management of MASLD and, similarly to dietary interventions, is recommended as a first-line intervention by most clinical guidelines. Regular exercise has been shown to improve insulin sensitivity, reduce hepatic fat accumulation, and lower markers of inflammation and oxidative stress—key drivers in MASLD progression [34]. Aerobic exercise, such as brisk walking, cycling, or swimming, is especially effective at reducing visceral adiposity and intrahepatic triglyceride content, with benefits observed even in the absence of significant weight loss. Studies have demonstrated that physical activity alone can decrease liver fat by 2% to 50%, depending on intensity and duration [35,36,37]. The most commonly recommended regimen includes moderate-intensity aerobic activity for 150–200 min per week, complemented by resistance training to preserve lean muscle mass and enhance metabolic health.

## 5. Pharmacological Treatment

Despite lifestyle modification remaining the cornerstone of MASLD management, pharmacotherapy is increasingly recognized as a critical adjunct, especially in patients with biopsy-confirmed metabolic steatohepatitis (MASH), significant fibrosis (≥F2), or coexisting cardiometabolic comorbidities. Several agents have demonstrated histological and biochemical effects by targeting core pathophysiological pathways: oxidative stress, insulin resistance, lipotoxicity, and inflammation. Although no drug is currently globally approved specifically for MASLD, some drugs are locally recommended or used off-label in patients diagnosed with liver steatosis (Table 1).

**1.**  1. **Pioglitazone (30–45 mg/day)**

Pioglitazone, a thiazolidinedione, acts as a selective peroxisome proliferator-activated receptor gamma (**PPAR-γ**) agonist. It promotes adipocyte differentiation and enhances peripheral insulin sensitivity, thereby reducing the flux of free fatty acids (FFAs) to the liver. Pioglitazone also decreases hepatic de novo lipogenesis and inflammatory cytokine expression while increasing adiponectin levels, which has anti-inflammatory and insulin-sensitizing properties. RCTs, including PIVENS, have shown histological improvements in steatosis, lobular inflammation, and hepatocellular ballooning in patients with MASH, especially those with T2DM [13,15,38,39,40]. However, its use may be limited by adverse effects such as weight gain and fluid retention [38,39,40].

**2.**  **Vitamin E (800 IU/day, d-α-tocopherol)**

Vitamin E is a lipid-soluble antioxidant that reduces oxidative damage within hepatocytes, a key driver in the progression from steatosis to steatohepatitis [15]. It neutralizes reactive oxygen species (ROS) and downregulates pro-inflammatory signaling pathways. The PIVENS trial demonstrated significant improvement in steatohepatitis but not fibrosis among non-diabetic adults with biopsy-proven NASH [38]. Despite moderate improvements in transaminases and histology, concerns remain about long-term safety, including a possible increased risk of prostate cancer in older men and hemorrhagic stroke [41,42]. Thus, it is not recommended in patients with diabetes or advanced cirrhosis.

**3.**  **Ursodeoxycholic Acid (UDCA, 13–15 mg/kg/day)**

UDCA is a hydrophilic bile acid with cytoprotective, anti-apoptotic, and anti-inflammatory properties. It stabilizes hepatocyte membranes, reduces hepatic transaminase levels in serum, and protects hepatocytes from oxidative stress [43,44,45]. While extensively used in cholestatic liver diseases, evidence for its efficacy in MASLD is limited and inconclusive. Some small trials have reported improvements in liver enzymes and steatosis, but histological benefits are uncertain [45,46,47,48]. It is worth noting that most of the evidence for UDCA use comes from patients with NASH or cholestatic liver diseases.

## 6. Review of New Drugs Currently Tested in RCTs

**Figure 3:**
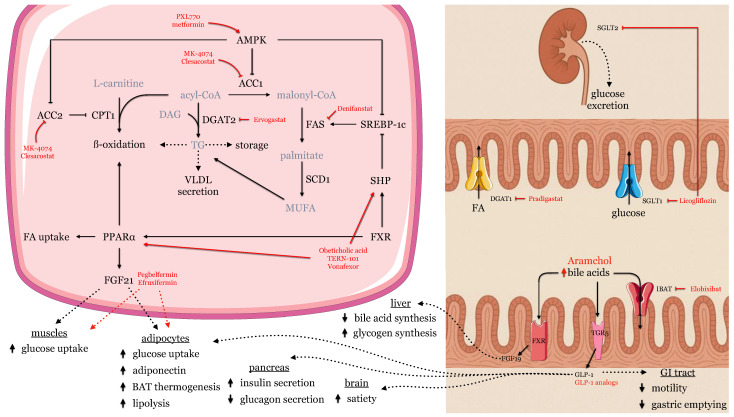
Main molecular effects of new, experimental drugs in MASLD.

In recent years, a growing number of investigational drugs have been studied, targeting various metabolic pathways involved in MASLD pathogenesis. The following section presents a review of contemporary RCTs, primarily phase 2 studies, evaluating the efficacy and safety of these novel therapeutic agents. The ClinicalTrials.gov database was searched up to April 2025 for clinical trials reporting a reduction in liver steatosis with any drugs used in the treatment of NAFLD/MASLD. Experimental therapies were grouped by their molecular mechanisms of action (Table 2, Figure 3).

## 7. Acetyl-CoA Carboxylase (ACC) Inhibitors

Acetyl-CoA carboxylase (ACC) plays a pivotal role in hepatic lipid metabolism by catalyzing the conversion of acetyl-CoA to malonyl-CoA, the first step in de novo lipogenesis. This process contributes to hepatic triglyceride accumulation, a hallmark of MASLD. ACC exists in two isoforms: ACC1, primarily involved in lipogenesis, and ACC2, which regulates fatty acid oxidation by inhibiting carnitine palmitoyltransferase 1 (CPT1). Inhibition of ACC reduces de novo lipogenesis and may enhance fatty acid oxidation, thereby decreasing hepatic steatosis [49,94].

In the NCT01431521 study, MK-4074, a dual ACC1/ACC2 inhibitor, administered at 200 mg twice daily for four weeks, resulted in a −36% reduction in intrahepatic fat, significantly outperforming pioglitazone [49]. The treatment was well tolerated and did not increase the rate of adverse events, although it had no effect on ALT or AST levels. Another ACC inhibitor, PF-05221304, was evaluated in several trials (NCT03248882, NCT03776175, and NCT04399538), either as monotherapy or in combination with diacylglycerol acyltransferases 2 (DGAT2) inhibitor PF-06865571 [50,51,52]. PF-05221304 showed a dose-dependent reduction in liver fat content—up to −40% in some regimens—and was also associated with reductions in ALT. However, its use led to elevations in serum triglycerides, a known adverse effect of ACC inhibition. Importantly, combining PF-05221304 with a DGAT2 inhibitor, PF-06865571, did not mitigate this triglyceride elevation. These findings highlight both the therapeutic potential and limitations of ACC inhibitors in MASLD, emphasizing the need for strategies that can offset the associated metabolic side effects.

## 8. Amino Acids, Carnitine, and N-Acetylcysteine

N-acetylcysteine and carnitine have been proposed as therapeutic agents in MASLD due to their roles in mitochondrial function, oxidative stress reduction, and metabolic regulation. N-acetylcysteine replenishes intracellular glutathione stores, reducing oxidative stress—a key driver of hepatocellular injury in MASLD [95,96]. Carnitine, on the other hand, is essential for mitochondrial fatty acid transport and β-oxidation, may enhance lipid metabolism, and mitigates hepatic steatosis [97,98,99].

These concepts were tested in the phase 2 clinical trial NCT04073368, which evaluated the safety and efficacy of two investigational amino acid-based formulations—AXA1125 and AXA1957—in patients with NASH [53]. AXA1125 includes leucine, isoleucine, valine, arginine, glutamine, and N-acetylcysteine, while AXA1957 contains leucine, isoleucine, arginine, glutamine, serine, and carnitine. Both formulations were designed to reduce inflammation and restore mitochondrial function. However, the addition of branched amino acids raises some concerns about the molecular effects of the tested formulations, since both leucine and isoleucine restriction were reported to ameliorate insulin resistance and NAFLD [100,101].

Nevertheless, over the treatment period, both AXA1125 and AXA1957 led to reductions in hepatic steatosis of −22.9% and −20.3%, respectively, and serum ALT levels of −21.9% and −20.6%, respectively, indicating a favorable impact on liver fat content and hepatocellular injury. These effects support the hypothesis that N-acetylcysteine and carnitine supplementation may help modulate oxidative stress and β-oxidation implicated in MASLD pathogenesis.

## 9. AMPK Activators

AMP-activated protein kinase (AMPK) serves as a central regulator of cellular energy homeostasis, influencing pathways related to lipid metabolism, glucose uptake, and inflammation [102,103]. Out of many downstream effects of AMPK activation, the most important in MASLD are inhibition of ACC, which promotes ß-oxidation; inhibition of sterol regulatory element-binding protein 1 (SREBP-1c), which downregulates fatty acid synthase (FAS) and de novo lipogenesis; and inhibition of β-Hydroxy β-methylglutaryl-CoA (HMG-CoA) reductase, which reduces cholesterol synthesis and promotion of glucose transporter 1 and 4 translocation towards cell membranes. Thus, AMPK activation has been proposed as a therapeutic strategy to mitigate hepatic steatosis and improve insulin sensitivity.

In the STAMP-NAFLD trial (NCT03763877), the direct AMPK activator PXL770 was evaluated in a randomized, double-blind, placebo-controlled phase 2a study involving 120 patients with hepatic steatosis [54]. Over 12 weeks of treatment, patients receiving PXL770 at 250 mg twice daily and 500 mg once daily achieved statistically significant reductions in liver fat content of −14.3% and −14.7%, respectively, as well as significant improvements in ALT, AST, HbA1c, and FIB-4 scores. These metabolic and hepatic benefits were observed without significant effects on lipid profiles or body weight, and the treatment was well tolerated. Separately, the NCT00063232 study evaluated metformin, an indirect AMPK activator, in patients with biopsy-confirmed NASH [55]. Treatment with 1 g of metformin twice daily for 48 weeks resulted in significant reductions in histological NASH activity, increased the number of patients with normal ALT levels, and improved insulin resistance as measured by HOMA-IR (−3.3 U). Collectively, these findings underscore the potential of AMPK-targeted therapies in MASLD, both in improving hepatic steatosis and modulating systemic metabolic dysfunction.

## 10. Bile Acid Metabolism

Bile acids are not only essential for lipid digestion but also act as signaling molecules regulating metabolic pathways through receptors like the farnesoid X receptor (FXR) and Takeda G protein-coupled receptor 5 (TGR5) [4,104]. Disruptions in bile acid homeostasis can contribute to hepatic steatosis, inflammation, and fibrosis. FXR activation in the liver suppresses lipogenic genes via indirect SREBP-1c inhibition and promotes fatty acid oxidation via PPARα, while in the intestine it regulates fibroblast growth factor 19 (FGF19), a hormone involved in bile acid synthesis feedback [105,106]. Although ileal bile acid transporter (IBAT) inhibition may reduce intestinal FXR signaling, increased hepatic bile acid production could enhance hepatic FXR activity, supporting improved lipid and glucose metabolism [107]. Additionally, elevated bile acid levels in the colon stimulate TGR5 receptors on enteroendocrine L-cells, leading to increased secretion of glucagon-like peptide-1 (GLP-1), which may provide additional effects in treatments targeting bile acid metabolism in MASLD [108].

Therapeutic strategies targeting bile acid metabolism, such as stearoyl-CoA desaturase 1 (SCD1) inhibition by aramchol and IBAT inhibition by elobixibat, aim to restore metabolic balance and mitigate liver damage in MASLD [109]. In the phase 2b ARREST trial (NCT02279524), 247 patients with biopsy-confirmed NASH received 400 mg or 600 mg of aramchol or placebo daily for 52 weeks [56]. Although the primary endpoint—a significant placebo-corrected reduction in liver triglyceride content—was narrowly missed (−3.1%; *p* = 0.066), notable secondary outcomes included NASH resolution without fibrosis worsening in 16.7% of patients in the 600 mg group versus 5% in the placebo group and fibrosis improvement in −29.5% compared to −17.5%. Aramchol also led to reductions in ALT (−17.3 U/L), AST (−10.8 U/L), and HbA1c (−0.13% points) and was well tolerated, supporting its role in modulating both hepatic inflammation and metabolic dysfunction.

Elobixibat (NCT04006145), by contrast, targets the IBAT disrupting enterohepatic circulation of bile acids and promoting hepatic bile acid synthesis. In a 16-week phase 2 trial involving 47 patients with NAFLD or NASH, daily elobixibat (5 mg) significantly lowered LDL cholesterol levels by −22 mg/dL on average. Despite the fact that the study did not assess its effect on liver steatosis, it supports the idea of targeting bile acid metabolism as a potential pharmacodynamic target in MASLD.

## 11. Diacylglycerol Acyltransferase Inhibitors

DGAT1 and DGAT2 catalyze the final step in triglyceride synthesis by esterifying diacylglycerol with acyl-CoA. DGAT2 is primarily active in hepatocytes, contributing to de novo lipogenesis and hepatic triglyceride accumulation, while DGAT1 functions in the intestine and peripheral tissues, facilitating dietary fat absorption and chylomicron creation. In MASLD, excessive activity of these enzymes leads to hepatic steatosis, lipotoxicity, and progression to steatohepatitis and fibrosis [110,111,112].

The NCT01811472 trial evaluated LCQ908, a DGAT1 inhibitor, in patients with hepatic steatosis and elevated triglycerides. Treatment led to a reduction in liver fat content of −2.9% and reduced ALT by −9.1 U/L, body weight by −2.5 kg, and waist circumference by −4.2 cm, while having no effect on AST, GGT, or serum triglyceride levels. The drug had a safety profile similar to placebo but was characterized by higher incident levels of diarrhea and abdominal pain.

In contrast, the DGAT2 inhibitor PF-06865571 was studied in NCT04399538 in combination with the ACC inhibitor clesacostat [52]. This trial included patients with a BMI between 25 and 40 kg/m^2^ and features of metabolic syndrome. The combination therapy resulted in a reduction in hepatic steatosis of up −60.3% but was associated with an increase in serum triglycerides of up to +27.9% and adverse events such as mild gastrointestinal disturbances and iron deficiency. Similarly, in the NCT03776175 trial, PF-06865571—administered alone or in combination with clesacostat—led to reductions in liver fat content of up to −40.1% in overweight or obese individuals [50].

## 12. Fatty Acid Synthase Inhibitors

Fatty acid synthase (FAS) is a key enzyme in the de novo lipogenesis pathway, responsible for the synthesis of palmitate. In MASLD, upregulated de novo lipogenesis contributes to hepatic steatosis and further lipotoxicity and inflammation. By inhibiting FAS, TVB-2640 reduces palmitate production, thereby decreasing the accumulation of toxic lipid species such as diacylglycerols and ceramides. This reduction in lipotoxic intermediates can alleviate hepatocellular stress, diminish inflammatory responses, and impede the activation of hepatic stellate cells, which are central to fibrogenesis [113,114].

The FASCINATE-1 trial (NCT03938246) evaluated TVB-2640, a first-in-class oral FASN inhibitor, in patients with NAFLD and stage 1–3 fibrosis [57]. In this 12-week phase 2a trial, patients with ≥8% liver fat and a BMI ≤ 40 kg/m^2^ received TVB-2640 at 25 mg or 50 mg daily or placebo. TVB-2640 significantly reduced liver fat in a dose-dependent manner, with the 50 mg group achieving a −28.1% reduction versus a +4.5% increase in the placebo group. Additionally, 61% of patients in the 50 mg group achieved a ≥30% liver fat reduction. The drug also decreased ALT levels by −20.5% and was well tolerated, with only mild adverse events reported.

## 13. Free Fatty Acid Receptor 4 Agonist

FFAR4, also known as GPR120, is a G-protein-coupled receptor activated by long-chain fatty acids, including omega-3 fatty acids. Activation of FFAR4 has been shown to exert anti-inflammatory effects by modulating macrophage polarization towards an anti-inflammatory M2 phenotype and inhibiting pro-inflammatory signaling pathways. In the context of MASLD, FFAR4 activation may reduce hepatic inflammation and fibrosis by attenuating inflammatory responses and improving insulin sensitivity [115,116].

The ICONA trial (NCT04052516) evaluated icosabutate, an oral free fatty acid receptor 1 and 4 (FFAR1/FFAR4) agonist, in patients with biopsy-confirmed MASH and fibrosis stages F1–F3 [58]. In this phase 2b study, 187 patients were randomized to receive 300 mg, 600 mg, or placebo daily for 52 weeks. Although the trial did not meet its primary endpoint—MASH resolution without fibrosis worsening—the 600 mg group showed a higher response rate than the placebo group (23.9% vs. 14.5%); however, the change was not statistically significant. Secondary outcomes included significant reductions in ALT (−30.1 U/L), AST (−18.5 U/L), GGT (−32.7 U/L), HOMA-IR (−3.8 U), bilirubin (−1.5 µM/L), hsCRP (−3.4 mg/L), and the fibrosis marker PRO-C3 (−11.8 µg/L). Fibrosis improvement of at least one stage occurred in 29.3% and 23.9% of patients receiving 300 mg and 600 mg, respectively, compared to 11.3% with placebo. However, no reduction in liver fat content was observed. These results suggest that while FFAR agonism may not directly reduce steatosis, it may provide antifibrotic and anti-inflammatory benefits in MASLD.

## 14. Fibroblast Growth Factor 21 Signaling

FGF21 is an endocrine hormone predominantly produced by the liver, playing a crucial role in regulating glucose and lipid metabolism. It exerts its effects by binding to fibroblast growth factor receptors (FGFRs), particularly FGFR1c, in complex with the co-receptor β-Klotho (KLB). Activation of this signaling pathway enhances insulin sensitivity, promotes fatty acid oxidation, reduces lipogenesis, and exerts anti-inflammatory effects [117,118].

The NCT03976401 trial assessed efruxifermin, a long-acting FGF21 analog, in patients with a BMI > 25 kg/m^2^ and hepatic fat content ≥ 10% [59,60,61]. Efruxifermin treatment led to a significant reduction in hepatic steatosis of −14.1% and reduced ALT levels by −32.3 U/L, liver stiffness by −37.6 kPa, and fibrosis biomarkers such as pro-C3 by −8.4 µg/L. However, gastrointestinal adverse events, including nausea, vomiting, and diarrhea, were reported. Another FGF21 analog, pegbelfermin (BMS-986036), was investigated in the NCT02413372 phase 2 trial involving patients with a BMI ≥ 25 kg/m^2^ [62,63]. Over a 16-week period, daily 10 mg or weekly 20 mg subcutaneous injections significantly reduced hepatic fat content (−6.8%) compared to placebo (−1.3%). The treatment was well tolerated, with only mild gastrointestinal symptoms reported. In contrast, NCT04583423 evaluated MK-3655, a humanized monoclonal antibody that activates FGFR1c/β-Klotho signaling by binding KLB [64]. This study included patients with NASH and a BMI between 25 and 50 kg/m^2^ but failed to demonstrate a significant therapeutic effect. Despite MK-3655’s lack of benefit, FGF21 analogs like efruxifermin and pegbelfermin show potential in improving steatosis, inflammation, and fibrosis in MASLD.

## 15. Farnesoid X Receptor Agonists

FXR is a nuclear receptor activated by bile acids, playing a pivotal role in regulating bile acid synthesis, lipid and glucose metabolism, and inflammatory response. FXR promotes ß-oxidation via PPARα activation and suppresses de novo lipogenesis via SREBP-1c inhibition by a small heterodimer partner (SHP) acting as a mediator. In the context of MASLD, FXR activation has been shown to reduce hepatic steatosis, inflammation, and fibrosis, making FXR agonists promising therapeutic agents [4,104].

Obeticholic acid (OCA), the most extensively studied FXR agonist, was evaluated in the FLINT trial (NCT01265498), where non-cirrhotic NASH patients receiving 25 mg daily for 72 weeks experienced notable improvements in liver histology, including reductions in steatosis, inflammation, and fibrosis [65,66,67,68]. Biochemical improvements included decreases in ALT (−38 U/L) and AST (−27 U/L). However, treatment was associated with pruritus and increases in alkaline phosphatase, HOMA-IR, and total and LDL cholesterol, without significant effects on triglycerides, HbA1c, waist circumference, or blood pressure. A separate phase 2 study (NCT00501592) in patients with NAFLD and T2DM demonstrated that 25–50 mg of OCA daily for six weeks reduced ALT (−9 U/L) and GGT (−39 U/L) [69].

Other FXR agonists under investigation include tropifexor, vonafexor, and EDP-305. Tropifexor (NCT04328077) showed dose-dependent reductions in ALT of up to −18%. Vonafexor (NCT03812029), evaluated in the LIVIFY trial, led to reductions in liver fat of up to −6.3% and reduced ALT by −16.3 U/L, GGT by −40.6 U/L, weight by up to −2.5 kg, and waist circumference by up to −2.2 cm [70]. EDP-305, a non-bile acid FXR agonist, at a 2.5 mg dose (NCT03421431) led to significant reductions in ALT of up to −26.1 U/L and liver fat of up to −6.4% [71]. Notably, EDP-305 also decreased ApoA1 levels, though it had no significant effects on lipid profiles or glycemic control.

Collectively, these studies highlight the capacity of FXR agonists to improve liver enzymes, steatosis, and fibrosis in MASLD. However, the consistent occurrence of pruritus and, in some cases, unfavorable changes in lipid profiles underscore the need for optimized dosing strategies and long-term evaluation to ensure both efficacy and tolerability.

## 16. GLP-1 and Dual GLP-1/GIP Agonists

GLP-1 receptor agonists enhance insulin secretion, suppress glucagon release, slow gastric emptying, and promote satiety, leading to weight loss and improved glycemic control. These effects contribute to reduced hepatic steatosis and inflammation and may provide a therapeutic alternative for MASLD patients [119,120].

Semaglutide, a GLP-1 receptor agonist, was evaluated in several phase 2 trials. In the NCT02970942 trial, patients with biopsy-proven NASH and fibrosis stages F1–F3 received daily subcutaneous injections for 72 weeks [74]. The highest dose (0.4 mg/day) led to NASH resolution without worsening of fibrosis in 59% of patients compared to 17% with placebo, along with significant reductions in steatosis scores, ALT (up to −60%), AST (up to −50%), GGT (up to −52%), HbA1c (up to 1.2% points) and body weight (up to −12.3 kg). However, in NCT03987451, where semaglutide was tested at a higher dose of 2.4 mg weekly in patients with compensated cirrhosis, it failed to produce significant improvements in fibrosis or NASH resolution, though beneficial effects on liver fat (−38%), liver enzymes, HbA1c (−1.4% points), body weight (−8.6 kg), and BMI (−3.1 units) were still observed [72,73].

Other trials reinforced the metabolic effects of semaglutide. In NCT04216589, conducted in patients with NAFLD and HIV, semaglutide led to reductions in steatosis (−4.2%), HbA1c, weight (−7.8 kg), BMI (−2.8 units), and waist circumference (−6.7 cm) [75]. Similarly, NCT04944992 demonstrated improvements in hepatic fat content (−42.3%), weight (−7.1 kg), and lipid parameters—including reductions in total cholesterol (−8.0%), LDL (−6.9%), triglycerides (−23.3%), and apoB (−9.8%), alongside an increase in HDL (+3.6%)—in overweight and obese patients with NAFLD [76].

In contrast, the SYNERGY-NASH trial (NCT04166773) investigated tirzepatide, a dual GLP-1/GIP receptor agonist, in patients with biopsy-confirmed MASH and fibrosis stages F2–F3 [77]. Weekly administration of tirzepatide at doses of 5, 10, and 15 mg for 52 weeks led to MASH resolution without worsening of fibrosis in up to 62% of patients and fibrosis improvement in over 50% of cases across all dose groups—substantially outperforming placebo. Tirzepatide also led to reductions in steatosis (−11.3%) and body weight (−17.9 kg).

Altogether, these studies illustrate that GLP-1 receptor agonists like semaglutide consistently improve steatosis, metabolic markers, and liver enzymes in MASLD, although effects on fibrosis may be more limited, particularly in advanced disease stages. Tirzepatide, through combined GLP-1 and GIP receptor activity, appears to offer superior efficacy in both NASH resolution and fibrosis regression, underscoring the potential of dual incretin-based therapies in MASLD management.

## 17. Dual GLP-1/Glucagon Receptor Agonists

Glucagon receptor activation plays a multifaceted role in hepatic metabolism. It promotes lipolysis, enhances basal energy expenditure, and influences liver lipid metabolism [121,122,123]. In the context of MASLD, glucagon receptor agonism may complement GLP-1 receptor agonism by directly stimulating hepatic fatty acid oxidation and reducing lipogenesis, leading to decreased hepatic steatosis. Moreover, glucagon receptor activation may attenuate hepatic inflammation and fibrosis, further contributing to its therapeutic potential in MASLD.

Dual GLP-1 and glucagon receptor agonists are an emerging class of therapeutics in MASLD that combine the metabolic benefits of GLP-1 receptor activation with the lipid-lowering and energy-expending effects of glucagon signaling. In the phase 2a trial NCT04944992, efinopegdutide was compared to semaglutide in patients with NAFLD [76]. Administered once weekly at a dose of 10 mg for 24 weeks, efinopegdutide produced a 72.7% reduction in liver fat content—significantly greater than the 42.3% reduction observed with semaglutide. In addition to reducing steatosis and body weight (−8.5 kg), efinopegdutide also led to decreases in total (−15.2%), LDL (−13.0 %), and HDL cholesterol (−8.1%); triglycerides (−30.9%); and apolipoprotein B (−14.7 %). These lipid-modifying effects suggest that glucagon receptor agonism enhances hepatic lipid metabolism beyond what is achieved with GLP-1 agonism alone.

In the NCT04771273 trial, survodutide—another dual GLP-1/glucagon receptor agonist—was evaluated in patients with biopsy-proven MASH and fibrosis stages F1–F3 [78]. After 48 weeks of treatment, survodutide was superior to placebo in achieving MASH resolution without fibrosis progression and demonstrating meaningful improvements in liver fat content (−13 %). The third agent in this class, cotadutide, was assessed in the NCT04019561 trial involving patients with NAFLD or NASH and fibrosis stages F1–F3 [79]. Over 19 weeks, cotadutide significantly reduced hepatic steatosis (−4.8%), ALT (−15.5%), AST (−14.0%), body weight (−2.9 kg), and BMI (−1.0 units).

Altogether, these trials highlight the potential of dual GLP-1/glucagon receptor agonists in treating MASLD through complementary mechanisms that reduce liver fat, improve cardiometabolic markers, and address liver inflammation. Their robust metabolic effects, alongside preliminary histological benefits, position this class as a promising option for future MASLD therapy.

## 18. Ketohexokinase Inhibition

Ketohexokinase (KHK), also known as fructokinase, is the key enzyme responsible for phosphorylating fructose to fructose-1-phosphate. The process promotes de novo lipogenesis and contributes to hepatic fat accumulation. In the context of MASLD, excessive dietary fructose intake and elevated hepatic KHK activity have been linked to increased triglyceride synthesis, steatosis, and insulin resistance [124,125]. Inhibiting KHK may therefore reduce hepatic lipid accumulation and improve metabolic parameters [126].

The phase 2a trials NCT03969719 and NCT03256526 evaluated PF-06835919, an oral KHK inhibitor, in 164 adults with NAFLD and T2DM [80,81]. Participants were randomized to receive 150 mg, 300 mg, or placebo daily for 16 weeks. Treatment with PF-06835919 led to dose-dependent reductions in liver fat content, with a 19% reduction in the 300 mg group and 17% in the 150 mg group, compared to only 5% in the placebo group. ALT levels also decreased, supporting a hepatoprotective effect. Although both active treatment arms showed significant reductions in HbA1c from baseline, these were not statistically significant versus placebo, and no significant changes were observed in HOMA-IR, suggesting a limited effect on insulin sensitivity. The drug was well tolerated, with mostly mild gastrointestinal symptoms and no serious adverse events reported.

## 19. Mitochondrial Uncoupling

Mitochondria serve as the principal sites of cellular energy production, generating adenosine triphosphate (ATP) via oxidative phosphorylation. This process involves the transfer of electrons through the mitochondrial electron transport chain (ETC), which drives the translocation of protons across the inner mitochondrial membrane, thereby establishing an electrochemical proton gradient. ATP synthase subsequently utilizes this gradient to catalyze the phosphorylation of ADP to ATP. Mitochondrial uncoupling refers to the dissipation of the proton gradient independent of ATP synthesis, typically through proton leak pathways that permit the re-entry of protons into the mitochondrial matrix. This uncoupling process reduces the efficiency of oxidative phosphorylation, resulting in increased substrate oxidation and energy expenditure, with excess energy released as heat. These effects can contribute to decreased lipid accumulation and improved metabolic homeostasis [127,128].

HU6, a controlled-release oral prodrug of 2,4-dinitrophenol (DNP), exerts its effects by selectively and mildly uncoupling oxidative phosphorylation. Unlike old uncouplers, which pose significant toxicity risks due to uncontrolled mitochondrial disruption, HU6 is designed to achieve a wider therapeutic window of uncoupling that safely enhances mitochondrial energy dissipation [129,130].

The phase 2a clinical trial NCT04874233 evaluated HU6, an oral prodrug of the mitochondrial uncoupler 2,4-dinitrophenol (DNP), in individuals with NAFLD [82]. In the study, 80 adults with a BMI of 28–45 kg/m^2^ and ≥8% liver fat were randomized to receive daily oral doses of HU6 (150, 300, or 450 mg) or placebo for 61 days [82]. HU6 significantly reduced liver fat content at all doses, with mean relative reductions of −26.8% (150 mg), −35.6% (300 mg), and −33.0% (450 mg), compared to a +5.4% increase in the placebo group.

These findings indicate that mitochondrial uncoupling via HU6 can effectively reduce hepatic steatosis and support weight loss in patients with MASLD. However, the treatment was associated with a slightly higher incidence of diarrhea and flushing.

## 20. Nonsteroidal Anti-Inflammatory Drugs

Acetylsalicylic acid (ASA)’s beneficial effects in MASLD are attributed to its anti-inflammatory and antiplatelet properties. It irreversibly inhibits cyclooxygenase enzymes (COX-1 and COX-2), leading to decreased synthesis of pro-inflammatory prostaglandins and thromboxanes. This inhibition reduces hepatic inflammation, which is implicated in the progression of liver steatosis to steatohepatitis and fibrosis. Furthermore, ASA has been shown to suppress the transforming growth factor-beta1 (TGF-β1)/Smad signaling pathway, which plays a crucial role in hepatic fibrogenesis. By attenuating this pathway, ASA may help prevent the development of liver fibrosis [131,132,133,134].

The phase 2 clinical trial NCT04031729 evaluated the efficacy of low-dose ASA (81 mg daily) over six months in adults with MASLD without cirrhosis [83]. This randomized, double-blind, placebo-controlled study demonstrated that ASA significantly reduced hepatic fat content compared to placebo. Specifically, participants receiving it experienced a mean absolute reduction in liver fat of −6.6%, whereas the placebo group showed a mean increase of +3.6%. Additionally, 43% of ASA-treated individuals achieved at least a 30% relative reduction in liver fat, compared to 13% in the placebo group.

## 21. PPAR Agonists

PPARs are nuclear hormone receptors that regulate gene expression involved in lipid metabolism and glucose homeostasis [13,38,39,40]. There are three isoforms: PPAR-α, PPAR-γ, and PPAR-δ. PPARs are key transcription factors that coordinate hepatic metabolism in response to nutritional cues. Among them, PPARα plays a central role in adapting liver function during feeding and fasting transitions. In the fasted state, PPARα is activated by fatty acids released from adipose tissue and promotes their hepatic uptake and mitochondrial β-oxidation, partly through transcriptional activation of transport proteins (e.g., CD36 and SLC27A1) and enzymes such as CPT1A. This pathway supports energy production and ketogenesis, while also inducing the hepatokine FGF21, which enhances systemic glucose and lipid metabolism. Additionally, PPARα activates autophagy (notably lipophagy), helping maintain energy balance. During the fed state, PPARα contributes to triglyceride synthesis by promoting acetyl-CoA transport and activating lipogenic regulators like SREBP1C. PPARγ and PPARδ also contribute, with PPARγ enhancing lipid storage via mTORC1 signaling and PPARδ boosting β-oxidation and HDL formation. Together, PPARs orchestrate lipid and glucose metabolism to maintain hepatic and systemic energy homeostasis.

Lanifibranor, a pan-PPAR agonist activating α, γ, and δ isoforms, was investigated in two trials (NCT03008070 and NCT03459079) [88,89,90,91]. In the phase 2b NATIVE trial (NCT03008070), 49% of patients receiving 1200 mg daily achieved NASH resolution without worsening fibrosis, compared to 27% with placebo, and 55% showed at least one-stage fibrosis improvement. Lanifibranor also significantly reduced ALT (−26.1 U/L), AST (−15.1 U/L), hsCRP (−2.0 mg/L), HOMA-IR (−5.8 units), HbA1c (−0.4% points), and triglycerides (−43.4 mg/dL). In the NCT03459079 trial, the drug additionally reduced hepatic steatosis (−8.7%) and HbA1c (−0.7% points), while increasing HDL cholesterol by +7.6 mg/dL.

Saroglitazar, a dual PPAR-α/γ agonist, was studied in the trials NCT03061721 and NCT03863574 [86,87]. In the NCT03061721, daily 4 mg dosing significantly reduced liver fat content (−4.2%), ALT levels (−44.9%), and fibrosis scores. The drug was characterized by a favorable safety profile. These findings were corroborated by NCT03863574, where saroglitazar led to reductions in ALT (−29.6 U/L), AST (−16.3 U/L), and GGT (−28.4 U/L).

Finally, dR-pioglitazone, a deuterium-stabilized R-stereoisomer of pioglitazone and selective PPAR-γ agonist, was evaluated in NCT04321343. This study demonstrated reductions in fibrosis scores, hepatic steatosis (up to −21.3%), ALT (up to −13.6 U/L), AST (up to −10.2 U/L), GGT (up to −21.8 U/L), HbA1c (up to −0.2% points), and HOMA-IR (up to −0.21 units), highlighting its potential for targeting insulin resistance and hepatic injury in patients with biopsy-proven MASLD [84,85].

## 22. SGLT1 and SGLT2 Inhibitors

Sodium-glucose co-transporter 1 and 2 (SGLT1/2) are key glucose transporters in the intestine and kidneys, and molecular targets of antidiabetic drugs, flozins. SGLT1 inhibition in the intestine reduces postprandial glucose absorption, while SGLT2 inhibition in the kidneys promotes urinary glucose excretion. Together, these effects reduce glucose and insulin levels, enhance fatty acid β-oxidation, lower insulin resistance, and promote caloric deficit, all relevant to MASLD pathophysiology [24,135,136].

The phase 2a clinical trial NCT03205150 investigated licogliflozin, a dual SGLT1/SGLT2 inhibitor [92]. In this randomized, placebo-controlled study, patients received either 30 mg or 150 mg of licogliflozin daily for 12 weeks. The 150 mg group showed significant reductions in ALT (−30.4 U/L) and improvements in hepatic steatosis (−6.9%) as measured by MRI-PDFF, along with reductions in AST (−17.0 U/L) and body weight (−4.5 kg). The treatment was generally well tolerated, though gastrointestinal side effects, especially diarrhea, were more frequent at the higher dose.

## 23. Testosterone

The therapeutic rationale for testosterone supplementation in MASLD is supported by growing evidence that low testosterone levels in men are associated with increased visceral adiposity, insulin resistance, and hepatic steatosis. Testosterone enhances insulin sensitivity by improving insulin signaling pathways and decreasing adipose tissue-derived inflammation [137]. Additionally, testosterone promotes hepatic fatty acid oxidation while downregulating de novo lipogenesis, contributing to a reduction in intrahepatic triglyceride accumulation. Its anti-inflammatory effects further help suppress hepatic production of pro-inflammatory cytokines and reduce oxidative stress, both of which play a role in the transition from simple steatosis to steatohepatitis and fibrosis [138,139,140].

The phase 2 LiFT trial (NCT04134091) evaluated LPCN 1144, an oral prodrug of testosterone, in men with biopsy-confirmed NASH and fibrosis stages F1–F3 [93]. In this 36-week, randomized, placebo-controlled trial, participants received LPCN 1144 either alone or in combination with vitamin E. Both treatment arms showed significant reductions in liver fat content by week 12, with mean absolute reductions of up to −9.2%. By week 36, both groups achieved statistically significant NASH resolution without worsening fibrosis. Additionally, treatment led to reductions in fibrosis, ALT (−22.9 U/L), AST (−12.0 U/L), ALP (−8.5 U/L), and GGT (−13.4 U/L). LPCN 1144 was well tolerated, with adverse event rates similar to placebo.

These findings suggest that testosterone replacement through LPCN 1144 may serve as a potential therapeutic approach in MASLD, particularly in hypogonadal men, by targeting hepatic steatosis, liver injury, and fibrosis. Further studies are needed to confirm long-term outcomes and clarify its role in broader patient populations.

## 24. THR-β Agonists

The therapeutic effects of thyroid hormone receptor beta (THR-β) agonists like TERN-501 in MASH are attributed to their ability to enhance hepatic lipid metabolism through mitochondrial biogenesis and β-oxidation. THR-β is predominantly expressed in the liver, where its activation promotes fatty acid oxidation and reduces hepatic lipogenesis—key mechanisms relevant to MASLD [141]. Unlike non-selective thyroid hormone therapies, THR-β agonists aim to provide metabolic benefits without systemic hormonal side effects.

The DUET trial (NCT05415722) evaluated TERN-501, a selective THR-β agonist, in patients with MASH. In this 12-week, randomized, double-blind, placebo-controlled phase 2a study, TERN-501 was administered at varying doses as monotherapy or in combination with the FXR agonist TERN-101. The primary endpoint was a change in liver fat content as measured by MRI-PDFF. TERN-501 monotherapy significantly reduced liver fat content across all dose groups, reaching −44.8% in the 6 mg group. The treatment was well tolerated, with only a slightly higher incidence of pruritus.

## 25. Conclusions

The growing body of evidence from recent RCTs underscores the remarkable diversity of molecular pathways implicated in MASLD pathogenesis. A wide array of investigational drugs has emerged, targeting key molecular mechanisms involved in disease progression. Among these, GLP-1 receptor agonists, PPAR agonists, and FXR agonists, as well as inhibitors of ACC and DGAT, have been the most extensively studied and demonstrate the most promising therapeutic potential to date. Notably, GLP-1 receptor agonists, as well as dual GLP-1/GIP and GLP-1/glucagon receptor agonists, appear to exert the most comprehensive effects on MASLD, simultaneously improving steatosis, insulin resistance, inflammation, and body weight. While several agents have shown encouraging results in terms of hepatic fat reduction, metabolic improvement, and even histological benefits, the majority of available data come from small, early-phase trials, primarily phase 2 studies. The current landscape therefore reflects an exciting but still preliminary stage of drug development in MASLD. Robust, large-scale phase 3 trials are still needed to confirm these findings, evaluate long-term safety and efficacy, and ultimately establish evidence-based pharmacologic treatments for widespread clinical use.

## Figures and Tables

**Table 1:** Contemporary pharmacological treatment of MASLD.

Drug	Dose	Mechanism of Action	Histological Effect	Notable Side Effects
**Pioglitazone** **[13,15,38,39,40]**	30–45 mg/day	PPAR-γ agonist; improves insulin sensitivity, reduces hepatic lipotoxicity	Improves steatosis, inflammation, and hepatocyte ballooning	Weight gain, fluid retention, possible increased risk of bladder cancer
**Vitamin E** **[15,38,41,42]**	800 IU/day	Antioxidant; neutralizes ROS, reduces oxidative stress and hepatocyte injury	Improves steatohepatitis, no effect on fibrosis	Possible increased risk of prostate cancer
**Ursodeoxycholic acid** **[43,44,45,46,47,48]**	13–15 mg/kg/day	Hydrophilic bile acid; cytoprotective, anti-inflammatory, reduces bile acid toxicity	Uncertain; a small number of studies show biochemical but not histological improvement	Diarrhea

**Table 2:** A summary of experimental drugs used in treatment of MASLD.

Name	Reference	NCT	Mechanism of Action	Duration/Phase	Results Posted	Effect	Population
**MK-4074**	**[49]**	**NCT01431521**	Dual acetyl-CoA carboxylase (ACC1 and -2) inhibitor	4 weeks, 1	2016	**↓** steatosis; **no effect**; AST, ALT	Obese, NAFLD
**Clesacostat PF-05221304**	**[50]**	**NCT03248882**	Dual acetyl-CoA carboxylase (ACC1 and -2) inhibitor	16 weeks, 2a	2020	**↓** steatosis, ALT	Overweight/obese, NAFLD, or NASH
**Clesacostat PF-05221304**	**[51]**	**NCT03776175**	Dual acetyl-CoA carboxylase (ACC1 and -2) inhibitor	6 weeks, 2a	2020	**↓** steatosis	Overweight/obese NAFLD, T2DM, or other metabolic syndrome comorbidities
**Clesacostat PF-05221304**	**[52]**	**NCT04399538**	Dual acetyl-CoA carboxylase (ACC1 and -2) inhibitor	6 weeks, 2a	2023	**↓** steatosis; **↑** TG	Overweight/obese NAFLD
**AXA1125 & AXA1957**	**[53]**	**NCT04073368**	Amino acids and NAC	16 weeks, N/A	2021	**↓** steatosis, ALT; **↑** AE; **no effect**: AST, HOMA-IR	NASH
**PXL770**	**[54]**	**NCT03763877**	AMPK activator	12 weeks, 2	2021	**↓** steatosis, ALT, AST, HbA1c, Fib4; **no effect**: lipid profile, weight	Overweight/obese NAFLD
**Metformin**	**[55]**	**NCT00063232**	AMPK activator	48 weeks, 2	2011	**↓** inflammation, ALT, HOMA-IR	NASH
**Aramchol**	**[56]**	**NCT02279524**	Bile salt fatty acid conjugate	52 weeks, 2b	2021	**↓** steatosis, fibrosis, ALT, AST, HbA1c	Overweight/obese NASH
**Elobixibat**		**NCT04006145**	Inhibitor of the ileal bile acid transporter	16 weeks, 2	2021	**↓** LDL	NAFLD/NASH
**Pradigastat LCQ908**		**NCT01811472**	DGAT1 Inhibitor	24 weeks, 2	2016	**↓** steatosis, ALT, weight, WC; **no effect:** AST, GGTP, TG	NAFLD
**Ervogastat PF-06865571**	**[51]**	**NCT03776175**	DGAT2 inhibitor	6 weeks, 2a	2020	**↓** steatosis	Overweight/obese NAFLD, T2DM, or other metabolic syndrome comorbidities
**Ervogastat PF-06865571**	**[52]**	**NCT04399538**	DGAT2 inhibitor	6 weeks, 2a	2023	**↓** steatosis; **↑** TG	Overweight/obese NAFLD
**Denifanstat TVB-2640**	**[57]**	**NCT03938246**	FAS inhibitor	12 weeks, 2	2024	**↓** steatosis, ALT	Overweight/obese NAFLD with stage 1-3 fibrosis
**Icosabutate**	**[58]**	**NCT04052516**	Free fatty acid receptor 4 (FFAR4) agonist	52 weeks, 2b	2025	**↓** ALT, GGTP, AST, HOMA-IR, bilirubin, hsCRP, pro-C3; **no effect:** steatosis	NASH
**Efruxifermin**	**[59,60,61]**	**NCT03976401**	FGF21 analog	12 weeks, 2a	2022	**↓** steatosis, ALT, liver stiffness, pro-C3	Overweight/obese, NAFLD
**Pegbelfermin BMS-986036**	**[62,63]**	**NCT02413372**	Pegylated FGF21	16 weeks, 2	2020	**↓** steatosis	Overweight/obese, NASH
**MK-3655**	**[64]**	**NCT04583423**	Humanized mAb that binds KLB FGFR1c/β-klotho activator	16 weeks, 2	2024	**No effect**	Overweight/obese, NASH
**Obeticholic acid INT-747**	**[65,66,67,68]**	**NCT01265498**	FXR agonist	72 weeks, 2	2015	**↓** histological steatosis, ALT, AST, GGTP, total bilirubin, HDL, body weight, BMI; **↑** alkaline phosphatase, HOMA-IR, total cholesterol, LDL; **no effect:** TG, HbA1c, WC, blood pressure	NASH
**Obeticholic acid INT-747**	**[69]**	**NCT00501592**	FXR agonist	6 weeks, 2	2012	**↓** ALT, GGTP	T2DM, NAFLD
**TERN-101**		**NCT04328077**	FXR agonist	12 weeks, 2	2022	**↓** ALT	Overweight/obese, NASH
**Vonafexor**	**[70]**	**NCT03812029**	FXR Agonist	12 weeks, 2a	2023	**↓** steatosis, ALT, GGTP, body weight, WC	F2/F3 fibrosis, NASH
**EDP-305**	**[71]**	**NCT03421431**	FXR agonist	12 weeks, 2	2021	**↓** steatosis, ALT, increase ApoA1; **no effect:** lipid profile, AboB, ApoC3, HOMA-IR, HbA1c	NASH
**Semaglutide**	**[72,73]**	**NCT03987451**	GLP-1 agonist	48 weeks, 2	2024	**↓** steatosis, ALT, AST, GGTP, HbA1c, HOMA-IR, body weight, BMI	Overweight/obese, NASH
**Semaglutide**	**[74]**	**NCT02970942**	GLP-1 agonist	72 weeks, 2	2021	**↓** steatosis, ALT, AST, GGTP, HbA1c, body weight	NASH
**Semaglutide**	**[75]**	**NCT04216589**	GLP-1 agonist	24 weeks, 2	2024	**↓** steatosis, HbA1c, HOMA-IR, body weight, BMI, WC	NAFLD, HIV infection
**Semaglutide**	**[76]**	**NCT04944992**	GLP-1 agonist	24 weeks, 2	2023	**↓** steatosis, weight, tot. chol. LDL, TG, apoB; **↑** HDL	Overweight/obese, NAFLD
**Tirzepatide**	**[77]**	**NCT04166773**	Dual GLP-1/GIP agonist	56 weeks, 2	2025	**↓** steatosis, fibrosis, body weight	Overweight/obese, NASH
**Efinopegdutide**	**[76]**	**NCT04944992**	Dual GLP-1/glucagon receptor agonist	24 weeks, 2	2023	**↓** steatosis, weight, tot. chol. LDL, TG, apoB, HDL	Overweight/obese, NAFLD
**Survodutide BI456906**	**[78]**	**NCT04771273**	Dual GLP-1/glucagon receptor agonist	48 weeks, 2	2024	**↓** steatosis, fibrosis	NASH or NAFLD with fibrosis stages F1-F3
**Cotadutide MEDI0382**	**[79]**	**NCT04019561**	Dual GLP-1/glucagon receptor agonist	23 days, 2	2022	**↓** steatosis, ALT, AST, body weight, BMI	Overweight/obese, NAFLD/NASH
**PF-06835919**	**[80,81]**	**NCT03969719**	Ketohexokinase inhibitor	16 weeks, 2a	2022	**↓** steatosis, ALT, HbA1c; **no change:** HOMA-IR	NAFLD, T2DM
**HU6**	**[82]**	**NCT04874233**	Mitochondrial uncoupler, prodrug of 2,4-dinitrophenol (DNP)	61 days, 2a	2024	**↓** steatosis	Overweight/obese, NAFLD
**Aspirin**	**[83]**	**NCT04031729**	Nonsteroidal anti-inflammatory drug (NSAID)	6 months,1/2	2024	**↓** steatosis	NAFLD
**dR-pioglitazone PXL065**	**[84,85]**	**NCT04321343**	Deuterium-stabilized R-stereoisomer of pioglitazone (PPARγ agonist)	36 weeks, 2	2023	**↓** steatosis, fibrosis, ALT, AST, GGTP, HbA1c, HOMA-IR	NAFLD and fibrosis score F1, F2, or F3
**Saroglitazar**	**[86]**	**NCT03061721**	Dual PPAR α/γ agonist	16 weeks, 2	2024	**↓** steatosis, fibrosis, ALT	Overweight/obese, NAFLD
**Saroglitazar**	**[87]**	**NCT03863574**	Dual PPAR α/γ agonist	24 weeks, 2	2024	**↓** ALT, GGTP, AST, TG	Overweight/obese, NASH
**Lanifibranor IVA337**	**[88,89,90,91]**	**NCT03008070**	Pan-PPAR agonist	24 weeks, 2	2021	**↓** fibrosis, ALT, AST, GGTP, hsCRP, HOMA, HbA1c, TG, ApoA1; **no effect:** total cholesterol and LDL; **↑** HDL, adiponectine	NASH
**Lanifibranor IVA337**	**[88,89,90,91]**	**NCT03459079**	Pan-PPAR agonist	24 weeks, 2	2024	**↓** steatosis, fibrosis, HbA1c, hepatic IR, fibrosis, **↑** HDL	NAFLD
**Licogliflozin LIK066**	**[92]**	**NCT03205150**	SGLT1 and SGLT2 inhibitor	12 weeks, 2	2021	**↓** steatosis, ALT, AST, body weight	NASH
**Lipocine LPCN 1144**	**[93]**	**NCT04134091**	Prodrug of testosterone	36 weeks, 2	2023	**↓** steatosis, fibrosis, ALT, AST, ALP, GGTP	NASH
**TERN-501**		**NCT05415722**	THR-β agonist	12 weeks, 2	2025	**↓** steatosis	Overweight/obese, NASH

## Data Availability

No new data were created or analyzed in this study. Data sharing is not applicable to this article.

## Abbreviations

The following abbreviations are used in this manuscript:

ACC	Acetyl-CoA carboxylase
ALP	Alkaline phosphatase
ALT	Alanine aminotransferase
AMPK	AMP-activated protein kinase
ASA	Acetylsalicylic acid
AST	Aspartate transaminase
ATP	Adenosine triphosphate
COX	Cyclooxygenase
CPT 1	Carnitine palmitoyltransferase 1
DGAT	Diacylglycerol acyltransferase
DNP	2,4-dinitrophenol
FAS	Fatty acid synthase
FFAR	Free fatty acid receptor
FXR	Farnesoid X receptor
FGF	Fibroblast growth factor
GIP	Gastric inhibitory polypeptide
GLP-1	Glucagon-like peptide-1
HMG-CoA	β-Hydroxy β-methylglutaryl-CoA
IBAT	Ileal bile acid transporter
KHX	Ketohexokinase
KLB	Co-receptor β-Klotho
MASLD	Metabolic dysfunction-associated steatotic liver disease
NAFLD	Non-alcoholic fatty liver disease
NASH	Non-alcoholic steatohepatitis
OCA	Obeticholic acid
PPAR	Peroxisome proliferator-activated receptor
RCT	Randomized clinical trial
SCD1	Stearoyl-CoA desaturase 1
SHP	Small heterodimer partner
SREBP-1c	Sterol regulatory element-binding protein 1
TGF-ß1	Transforming growth factor-beta1
TGR	Takeda G protein-coupled receptor
THR-ß	Thyroid hormone receptor ß
